# Allopurinol and 5-aminosalicylic acid influence thiopurine-induced hepatotoxicity in vitro

**DOI:** 10.1007/s10565-015-9301-1

**Published:** 2015-04-28

**Authors:** Mark M. T. J. Broekman, Hennie M. J. Roelofs, Dennis R. Wong, Mariska Kerstholt, Alex Leijten, Frank Hoentjen, Wilbert H. M. Peters, Geert J. A. Wanten, Dirk J. de Jong

**Affiliations:** Department of Gastroenterology, Radboud University Nijmegen Medical Center, 455, P.O. Box 9101, 6500 HB Nijmegen, The Netherlands; Department of Clinical Pharmacy & Toxicology, Orbis Medical Center, Sittard-Geleen, The Netherlands

**Keywords:** 5-Aminosalicylic acid, Allopurinol, 6-Mercaptopurine, Thioguanine, Azathioprine, HepaRG, HepG2, Huh7, Cytotoxicity, Thiopurines

## Abstract

**Introduction:**

The use of thiopurines is frequently accompanied by hepatotoxicity. Studies on hepatocyte cultures showed a time- and dose-dependent increase of thiopurine toxicity. 5-Aminosalicylic acid (5-ASA) and allopurinol can influence thiopurine metabolism; however, it is unknown whether this affects in vitro cytotoxicity.

**Methods:**

Human hepatoma cells (Huh7, HepG2 and HepaRG) were incubated with increasing concentrations of thiopurines, 5-ASA or allopurinol. Water-soluble tetrazolium salt-1 (WST-1) cytotoxicity assays were used to calculate cell survival curves and half maximal inhibitory concentrations (IC_50_). Combination experiments with thiopurines with a fixed dose of 200 μM 5-ASA or 100 μM allopurinol were conducted in HepaRG cells. Caspase-3/7 activation was evaluated, and single cell electrophoresis analysis was performed.

**Results:**

A time- and dose-related cytotoxic effect was seen with azathioprine (AZA) in all hepatoma cells, whereas Huh7 and HepG2 cells did not show toxicity to 6-mercaptopurine (6-MP). HepaRG cells expressed the highest levels of drug metabolising enzymes, and therefore, combination experiments were conducted in HepaRG cells. Addition of a non-toxic dose of allopurinol resulted in a twofold to threefold increased cytotoxicity of all thiopurines, which seemed to be mediated by apoptosis/DNA damage.

**Conclusion:**

The addition of allopurinol to thiopurines leads to a two–threefold increased cytotoxicity in HepaRG cells.

## Introduction

**Fig. 1:**
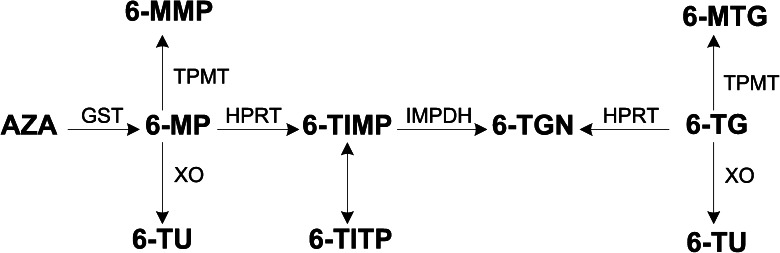
Simplified overview of thiopurine metabolism. Azathioprine (*AZA*) is converted by glutathione S-transferase (*GST*) in 6-mercaptopurine (*6-MP*). 6-MP can be methylated by thiopurine S-methyl transferase (*TPMT*) into 6-methyl mercaptopurine (*6-MMP*), oxidised by xanthine oxidase (*XO*) into 6-thiouric acid (*6-TU*) or metabolised by hypoxanthine-guanine phosphoribosyltransferase (*HPRT*) into 6-thioinosine monophosphate (*6-TIMP*). Next, 6-TIMP can be phosphorylated into 6-thioinosine triphosphate (*6-TITP*) or metabolised into the pharmacological active 6-thioguanine nucleotides (*6-TGN*). Besides metabolised into 6-TGN by HPRT, 6-thioguanine (*TG*) can be oxidised by XO into 6-TU or methylated by TMPT into 6-methyl thioguanine (*6-MTG*)

The metabolism of azathioprine (AZA) and 6-mercaptopurine (6-MP) is complex and involves multiple enzymatic steps, before the pharmacologically active substrate 6-thioguanine nucleotide (6-TGN) is produced (see Fig. 1). During this process, side products such as 6-methylmercaptopurine ribonucleotides (6-MMP) and thiouric acid are also formed. The metabolism of thioguanine (TG) is less complex with the main pathway resulting in a more direct conversion to 6-TGN. Several factors may influence thiopurine metabolism such as genetic variants in the thiopurine-S-methyltransferase (TPMT) gene and the use of concomitant drugs (Sahasranaman et al. 2008).

With respect to inflammatory bowel disease (IBD), the most relevant drugs that can influence thiopurine metabolism are 5-aminosalicylic acid (5-ASA) and allopurinol. Allopurinol, a strong xanthine oxidase (XO) and TPMT inhibitor, reduces 6-MMP formation (Blaker et al. 2013). The combination of allopurinol with a tailored dosage of AZA or 6-MP is a potent strategy in patients with a skewed metabolism towards the cytotoxic 6-MMP formation. Hoentjen et al. showed a significant reduction of 6-MMP levels from 10,110 pmol/8 × 10^8^ red blood cell (RBC) with monotherapy to 265 pmol/8 × 10^8^ RBC with combination therapy and significant increase of 6-TGN levels from 145 to 271 pmo1/8 × 10^8^ RBC (Hoentjen et al. 2013). Thiopurines and 5-ASA exert their disease-modifying effect by different mechanisms, yet 5-ASA can interfere in thiopurine metabolism by a non-competitive inhibition of TPMT (Szumlanski and Weinshilboum 1995). Therefore, co-administration can lead to a decreased production of 6-MMP and increase of the therapeutic active 6-TGN (Lowry et al. 2001; Dewit et al. 2002; de Graaf et al. 2010).

Studies to explore hepatotoxicity in humans on a cellular level are costly and have major ethical considerations. In vitro studies with primary cultures of hepatocytes or hepatoma cell lines are an ethical and feasible alternative for exploring drug toxicity. Previous in vitro studies demonstrated a time- and dose-dependent toxicity of AZA, 6-MP and 6-TG in HepaRG and HepG2 cells (Petit et al. 2008; de Vries et al. 2012). In this study, we aim to explore the effects of the addition of 5-ASA or allopurinol on thiopurine-induced hepatotoxicity.

## Materials and methods

HepG2 and Huh7 cells have been used extensively in in vitro experiments so far; therefore, we tested cytotoxicity in these hepatoma cell lines as well as in the more recently developed HepaRG cell line (Andersson et al. 2012). First, we assessed individual cytotoxicity of AZA, 6-MP and TG in all three cell lines. Next, we tested these cell lines for the expression of important drug metabolising enzymes and used the hepatoma cell line with the highest expression of drug metabolising enzymes to explore the influence of 5-ASA and allopurinol on cytotoxicity of thiopurines.

### Cell culture

Hepatocyte-differentiated HepaRG cells (Invitrogen™, Paisly, UK), HepG2 (HB-8065™, American Type Culture Collection, Rockville, USA) and Huh7 cells (JCRB0403, Cell Resource Center for Biomedical Research, Tohoku University, Japan) were used and cultured as described previously (Parent et al. 2004; de Vries et al. 2012). Cells were seeded into 96-well flat bottom microplates at a density of 1.48 × 10^5^ cells/cm^2^ for the HepG2 and Huh7 cells and 4.5 × 10^5^ cells/cm^2^ for the HepaRG cells according to the manufacturer’s protocol. After 24-h incubation, the medium was replaced by differentiation medium, being growth medium supplemented with 2.0 % (*v*/*v*) dimethyl sulfoxide (DMSO) (Sigma-Aldrich B.V.). Addition of DMSO is according to the protocol of growing HepaRG cells. To increase uniformity of the experiments, we also used differentiation medium in HepG2 and Huh7 cell cultures.

### Incubations with drugs

On the fourth day after seeding, cells were incubated with one of the following test substances; AZA, 6-MP, 5-ASA, allopurinol (all from Sigma-Aldrich B.V.) or TG (Alfa Aesar GmbH & Co KG, Karlsruhe, Germany) in concentrations ranging from 0.5 μM to 4 mM. For the combination experiments, a fixed non-toxic dose of 200 μM 5-ASA or 100 μM allopurinol was used in combination with the same range of thiopurines for 24, 48 or 72 h. In order to mimic daily drug administration, medium with drugs was refreshed every 24 h.

### Evaluation of cytotoxicity

Water-soluble tetrazolium salt-1 (WST-1) assays (Roche Diagnostics Nederland BV, Almere, the Netherlands) were performed after 24, 48 or 72 h of incubation with the drugs. The WST assay is based on the ability of viable cells to cleave the tetrazolium salt WST, which was measured with a Tecan Infinite m200PRO plate reader (Tecan, Giessen, the Netherlands) as described previously (de Vries et al. 2012). Three independent experiments were conducted in triplicate.

### Evaluation of drug metabolising enzymes

Protein expression of important drug metabolising enzymes was measured for two purposes: first to select the cell line with the highest expression (and therefore best comparability with human hepatocytes) and second to quantify any influence of adding DMSO to the culture medium of HepG2 and Huh7 cells. The following enzymes were analysed: UDP-glucuronosyltransferase 1A (UGT1A), cytochrome-P450 3A4 (CYP3A4), glutathione S-transferase alpha (GSTAlpha), mu (GSTMu) and theta (GSTTheta) (Eklund et al. 2006). These enzymes were chosen because of their role in thiopurine metabolism (GSTAlpha and GSTMu) or their important role in drug metabolism in general. Cells were seeded in 25 cm^2^ flasks, and after 24 h of culturing, half of the flasks were supplemented with differentiation medium (group DMSO+), while the other half received standard William’s E growth medium (group DMSO−). On days 0, 3 and 7, cells were harvested and homogenised using lysis buffer, consisting of 0.25 M saccharose, 20 mM Tris–HCl pH 7.4, 1 mM dithiothreitol (DTT) and 1 % Triton X-100 and lysates frozen at −20 °C until use. Total amount of protein was assessed according to the Lowry procedure (Lowry et al. 1951). Homogenates (25 μg protein) were loaded on 8, 10 or 12 % SDS polyacrylamide gels for UGT, CYP3A4 or GST analyses, respectively. After electrophoresis, proteins were transferred to nitrocellulose (Whatman GMBH, Dassel, Germany) using a semi-dry blotter (V20-SDB, Scie-Plas, Cambridge, UK). Non-specific binding was blocked with 1 % gelatine in phosphate-buffered saline (PBS)/Tween 20 (0.05 %, *v*/*v*). The following primary monoclonal antibodies were added: CYP3A4 (kind gift from Dr. P. Kremer, Université de Liège, Belgium), GST class Theta T1-1 (purchased from Dr. E. Juronen, Tartu, Estonia), UGT1A, GSTAlpha (A1-2) and GSTMu (all developed in our laboratory) (Peters et al. 1987; Peters et al. 1990; Peters et al. 1992). β-Actin (1:10,000, Sigma-Aldrich B.V.) was used as protein loading control. After incubation and washing, polyclonal rabbit anti-mouse peroxidase-conjugated second antibodies (Dako Diagnostics, Glostrup, Denmark) were added. Immunoreactive proteins were visualised with 0.1 % (*w*/*v*) 3,3-diaminobenzidine, 5 mM imidazole, 2 mM cobalt chloride hexahydrate and 8 μL 30 % hydrogen peroxide in 50 mL PBS.

### TPMT enzyme activity and polymorphism

To exclude influence caused by variation in TPMT activity, we analysed TPMT polymorphisms as well as TPMT enzyme activity. TPMT enzyme activity was assessed by high-performance liquid chromatography (HPLC) as described previously (Ford and Berg 2003). Genotyping of the three common variants in the *TPMT* gene (TPMT*2, *3A and *3B) was performed according to the manufacturer’s protocol using Taqman SNP genotyping assays (Life Technologies, Bleiswijk, the Netherlands, (TPMT*2: rs1800462, assay ID C__12091552_30; TPMT*3B: rs1800460, assay ID C__30634116_20; TPMT*3A: rs1142345, assay ID C__19567_20). Signals were detected with 7500 Fast Real-Time PCR system (Life Technologies) and subsequently analysed using the Allelic Discrimination software version 1.4 (Life Technologies).

### Thiopurine metabolite assessment

6-MMP and 6-TGN were assessed in cell lysates by a HPLC method as described by Dervieux et al. (Dervieux and Boulieu 1998). Metabolite analysis was only done for AZA, since the pharmacological action is based on the release of 6-MP. We assumed that if AZA is intracellular metabolised, 6-MP will be too. For this purpose, HepaRG cells were incubated with 600 μM AZA for 10 h; subsequently, the cells were washed with PBS 0.9 %, harvested, homogenised and stored at −20 °C until use.

### Caspase-3/7 activation

Caspase-3/7 activation was tested in HepaRG cells treated with AZA with and without 5-ASA or allopurinol with the Apo-ONE® Homogeneous Caspase-3/7 Assay (Promega) according to the manufacturer’s protocol. Briefly, HepaRG cells were seeded in a flat-bottom 96-well plate in a density of 20,000 cells/well. Cells were incubated with only medium (control), AZA (70 μM), 5-ASA (200 μM), allopurinol (100 μM), a combination of AZA + 5-ASA, a combination of AZA + allopurinol or staurosporine (2 μM) (positive control). After 24 h, Apo-one reagent was added. Caspase-3/7 activation was measured after 6 h on a Tecan Infinite m200PRO plate reader (Tecan, Giessen, the Netherlands). Two experiments were performed in triplicate. The control was set as reference to calculate differences in caspase-3/7 activation.

### Comet assay

The comet assay was used to identify the mechanisms behind the increased toxicity with allopurinol co-administration. This assay was performed according to the manufacturer’s instructions (Trevigen Inc., Gaithersburg, MD). HepaRG cells were seeded in 25-cm^2^ flasks and treated 24 h with allopurinol (100 μM), AZA (70 μM), AZA (70 μM) + allopurinol (100 μM) or untreated (control). After 24 h, cells were harvested and the cell suspension was mixed with 75 μL low melting agarose (Trevigen Inc.) in a density of 2 × 10^4^ cells/mL and directly pipetted on agarose-precoated slides. Slides were stored at 4 °C for 30 min and subsequently submerged in lysis solution. After 60-min lysis, they were treated with alkaline unwending solution (pH > 13) for 60 min, followed by 30-min electrophoresis at 25 V. Slides were stained with SYBR green (Trevigen Inc.) and visualised and photographed by a digital camera (AxioCam MRm) attached to a fluorescent microscope (Axio imager.M1) using a 20× magnification.

### Statistics

For the cytotoxicity analyses, the mean absorption of the empty wells was used to correct for background staining. The mean of three experiments was used for further calculations. Cell survival (in percentage of control) was calculated by dividing the mean of each concentration by the mean of the control wells (cells without drugs). Half maximal inhibitory concentrations (IC_50_) were calculated and subsequently tested among experiments using the extra sum of squares *F* test. Differences in caspase-3/7 activation were compared means of the by Mann–Whitney *U* test. Analyses were performed with GraphPad Prism (version 5.03 for Windows, GraphPad Software, San Diego, California, USA; www.graphpad.com).

## Results

### Single-drug tests

**Fig. 2:**
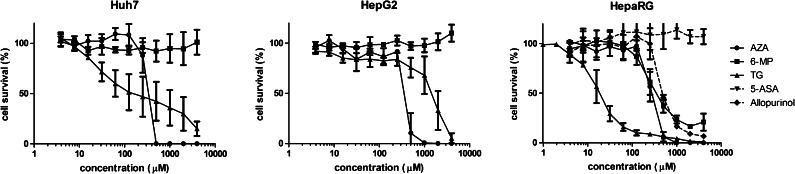
Cell survival after incubation of Huh7, HepG2 and HepaRG cells with azathioprine (*AZA*), 6-mercaptopurine (*6-MP*) or thioguanine (*TG*) for 72 h. In the HepaRG cells, 5-aminosalicylic acid (*5-ASA*) and allopurinol were also tested. Values are means with SEM from three independent experiments, performed in triplicate. *SEM* standard error of the mean

AZA showed a steep decline in cell survival at concentrations above approximately 200 μM in all cell lines (Fig. 2), with a time-dependent effect (Table 1). With respect to 6-MP, cell survival was not affected in the HepG2 and Huh7 cells; however, in the HepaRG cells, a decline in cell survival was seen when incubating for 48 or 72 h (Table 1). Incubation with TG resulted in a dose-dependent decline of cell survival observed in all cell lines, with the most pronounced decline in HepaRG cells. The lowest IC_50_ value (i.e. most cytotoxicity) was found in the HepaRG cells incubated for 72 h with TG, with an IC_50_ of 19 μM (Table 1).

**Table 1:** IC_50_ values in HepG2, HepaRG and Huh7 cells after 24, 48 or 72 h exposure to thiopurines, 5-ASA or allopurinol

		24 h	48 h	72 h
		IC_50_ (95 % CI)	IC_50_ (95 % CI)	IC_50_ (95 % CI)
HepG2	AZA	572 (520–628)	406 (333–495)**	363 (315–419)
	6-MP	n.r.	n.r.	n.r.
	TG	n.r.	1746 (1258–2422)**	1134 (812–1585)
Huh7	AZA	464 (431–501)	400 (314–509)	307 (188–501)
	6-MP	n.r.	n.r.	n.r.
	TG	2069 (856–5004)	172 (105–280)**	242 (120–486)
HepaRG	AZA	514 (460–574)	317 (289–347)**	266 (237–298)*
	6-MP	n.r.	679 (502–920)	412 (321–530)
	TG	1949 (993–3827)	100** (80–125)	19** (16–22)
	5-ASA	n.r.	n.r.	n.r.
	Allopurinol	n.r.	999 (766 – 1302)**	509 (411 – 631)**

Values are expressed in μM with 95 % confidence interval. Significantly different (*P* < 0.05)* or (*P* < 0.01)** compared to 24-h shorter incubation

*AZA* azathioprine, *6-MP* 6-mercaptopurine, *TG* thioguanine, *5-ASA* 5-aminosalicylic acid, *IC*_*50*_ half maximal inhibitory concentrations, *n.r.* IC_50_ not reached, *95 % CI* 95 % confidence interval

### Expression of drug metabolising enzymes

**Fig. 3:**
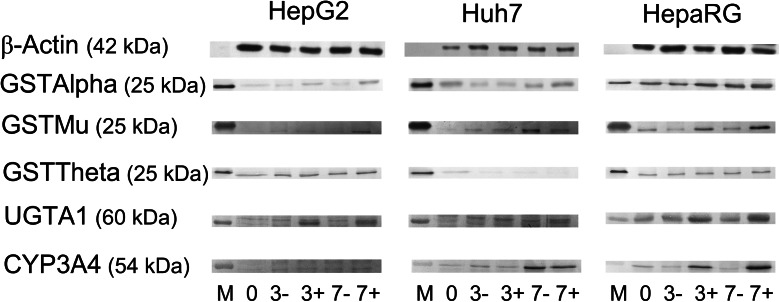
Expression of *β-actin*, *GSTAlpha*, *GSTMu*, *GSTTheta*, *CYP3A4* and *UGT1A* in *HepG2*, *Huh7* and *HepaRG* cells at day 0, after 3 days of incubation with William’s E medium without DMSO (3−) or William’s with DMSO 2 % (3+) and after 7 days of incubation without (7−) and with (7+) DMSO. *M* rainbow marker (Precision Plus Protein™ Kaleidoscope™) or antigen for the specific antibody (GST Alpha, Mu and Theta). *GSTAlpha* glutathione S-transferase alpha, *GSTMu* glutathione S-transferase Mu, *GSTTheta* glutathione S-transferase Theta, *CYP3A4* cytochrome-P450 3A4, *UGT1A* UDP-glucuronosyltransferase 1A, *DMSO* dimethyl sulfoxide

Large differences were seen between the three cell lines regarding the expression of UGT1A, CYP3A4, GSTAlpha, GSTMu and GSTTheta (Fig. 3). Huh7 and HepaRG cells showed CYP3A4 expression, whereas HepG2 cells did not. The HepaRG cells showed the highest expression of GSTAlpha and GSTMu. GSTTheta was expressed in both HepaRG and HepG2 cells but not at all in Huh7 cells. The latter was confirmed by PCR analysis, which showed a *GSTT1* gene deletion in Huh7 cells (data not shown). In HepG2 and Huh7 cells, addition of DMSO to the culture medium increased the expression of GST and UGTA1 enzymes. Overall, HepaRG cells showed the highest expression of all enzymes.

### Combination tests of thiopurines with 5-ASA or allopurinol

**Fig. 4:**
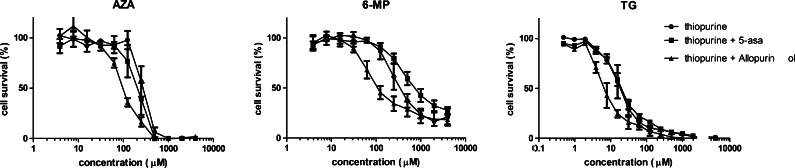
Cell survival curves after incubation of HepaRG cells with AZA, 6-MP or TG for 72 h with and without a fixed non-toxic concentration of 200 μM 5-ASA or 100 μM allopurinol. Values are means with SEM from three independent experiments, performed in triplicate. *AZA* azathioprine, *6-MP* 6-mercaptopurine, *TG* thioguanine, *5-ASA* 5-aminosalicylic acid, *SEM* standard error of the mean

Based on the highest expression of drug metabolising enzymes, HepaRG cells were used for the combination experiments with 5-ASA and allopurinol. 5-ASA did not influence cell survival of HepaRG cells at all, while incubation with allopurinol gave a decline in cell survival after 48 and 72 h with concentrations above 300 μM (Fig. 2 and Table 1). As can be seen in Fig. 4 and Table 2, incubation of thiopurines in combination with a fixed, non-toxic dose of 100 μM allopurinol had a larger influence on cytotoxicity than concomitant incubation with 200 μM 5-ASA. Co-administration of allopurinol with all thiopurines consistently resulted in an increased cytotoxicity, with the most pronounced cytotoxic effects emerging after 48 or 72 h. The combination of 6-MP and 5-ASA but not AZA and 5-ASA showed an increase in IC_50_ (i.e. decreased cytotoxicity) after 48 or 72 h incubation.

**Table 2:** IC_50_ values in HepaRG cells after 24, 48 and 72 h exposure to thiopurines with and without 200 μM 5-ASA or 100 μM allopurinol

	24 h			48 h			72 h		
	Single drug	+5-ASA	+Allopurinol	Single drug	+5-ASA	+Allopurinol	Single drug	+5-ASA	+Allopurinol
	IC_50_ (95 % CI)	IC_50_ (95 % CI)	IC_50_ (95 % CI)	IC_50_ (95 % CI)	IC_50_ (95 % CI)	IC_50_ (95 % CI)	IC_50_ (95 % CI)	IC_50_ (95 % CI)	IC_50_ (95 % CI)
AZA	514 (460–574)	538 (474–611)	502 (465–542)	317 (289–347)	271 (235–312)	201^ (168–241)	266 (237–298)	200* (167–239)	101^ (88–116)
6-MP	n.r.	n.r.	n.r.	679 (502–920)	n.r.*	403* (257–632)	412 (321–530)	918* (753–1118)	170^ (123–236)
TG	1949 (993–3827)	n.r. *	987* (730 1335)	75 (59–94)	100 (80–125)	68 (48–95)	19 (16–22)	21 (19–24)	9^ (7–11)

Values are expressed in μM with 95 % confidence interval. Combination experiments were similar to the single-drug experiments but with the addition of 200 μM 5-ASA or 100 μM allopurinol. Significantly different (*P* < 0.05)* or (*P* < 0.0001)^ compared to the same experiment without 5-ASA or allopurinol

*AZA* azathioprine, *6-MP* 6-mercaptopurine, *TG* thioguanine, *5-ASA* 5-aminosalicylic acid, *IC*_*50*_ half maximal inhibitory concentrations, *n.r.* IC_50_ not reached, *95 % CI* 95 % confidence interval

### TPMT activity and genotype

No mutations were found for the three most common allele variants in TPMT. TPMT activity was expressed as nmol 6-methylthioguanine/mg protein per hour and was lowest in HepG2 cells (0.08 nmol/mg protein per hour), followed by HepaRG cells (0.28 nmol/mg protein per hour) and Huh7 cells (0.47 nmol/mg protein per hour). Values were comparable with these found in other cell lines (Karim et al. 2013). TPMT activity was not correlated with the cytotoxicity.

### Metabolite formation

**Fig. 5:**
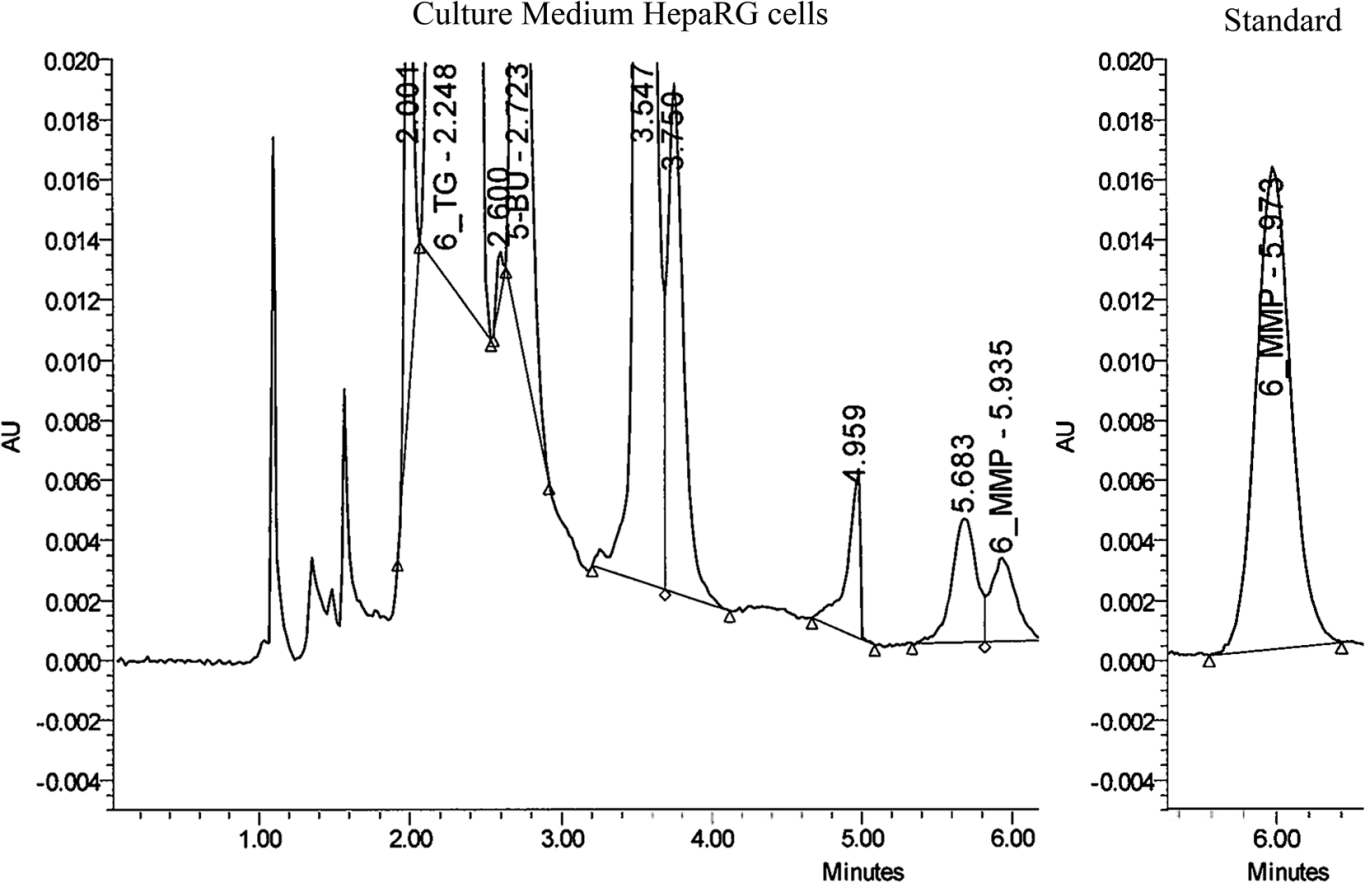
Formation of 6-MMP (RT: 5.935) metabolites, measured by HPLC in culture medium of HepaRG cells after incubation with AZA for 10 h. The RT of AZA and 6-MP is equal to that of 6-TGN; therefore, only 6-MMP metabolites could be distinguished. Other peaks are substances of culture medium. *6-MMP* 6-methylmercaptopurine, *RT* retention time, *AZA* azathioprine, *6-MP* 6-mercaptopurine, *6-TGN* thioguanine nucleotide

Support for in vitro metabolism of the thiopurines was obtained by HPLC. The retention time of 6-TGN was comparable to AZA and 6-MP; therefore, only 6-MMP metabolites could be identified by HPLC. In the culture medium of HepaRG cells incubated with AZA, we demonstrated the presence of 6-MMP metabolites, providing evidence for in vitro metabolism of AZA in HepaRG cells (Fig. 5).

### Mechanisms behind the increased cytotoxicity of allopurinol co-administration

**Fig. 6:**
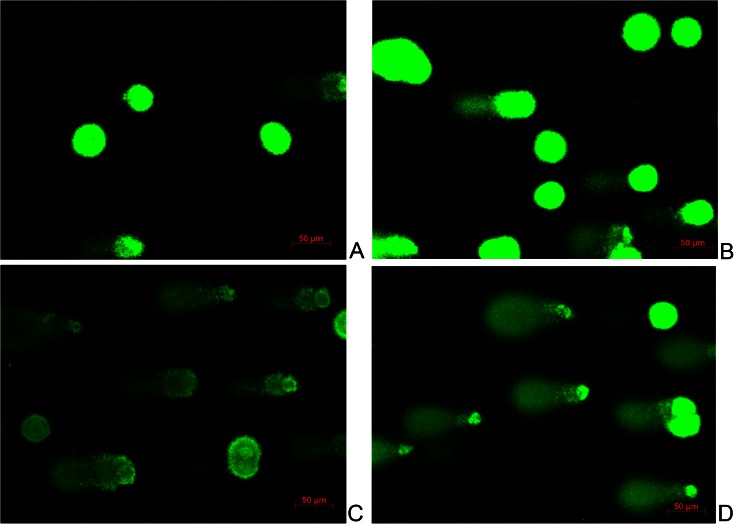
Single cell gel electrophoresis (Comet assay) of HepaRG cells incubated with **a** medium serving as control, **b** allopurinol (100 μM), **c** AZA (70 μM) and **d** AZA 70 (μM) and allopurinol (100 μM). HepaRG cells were embedded in low melting agarose on a slide, then lysed and treated with alkaline for DNA unwending followed by alkaline electrophoresis (pH > 13). Following SYBR Green I staining, nuclei were visualised under an epifluorescence microscope. Damaged DNA migrates towards the anode, resulting in a comet tail. *AZA* azathioprine

**Fig. 7:**
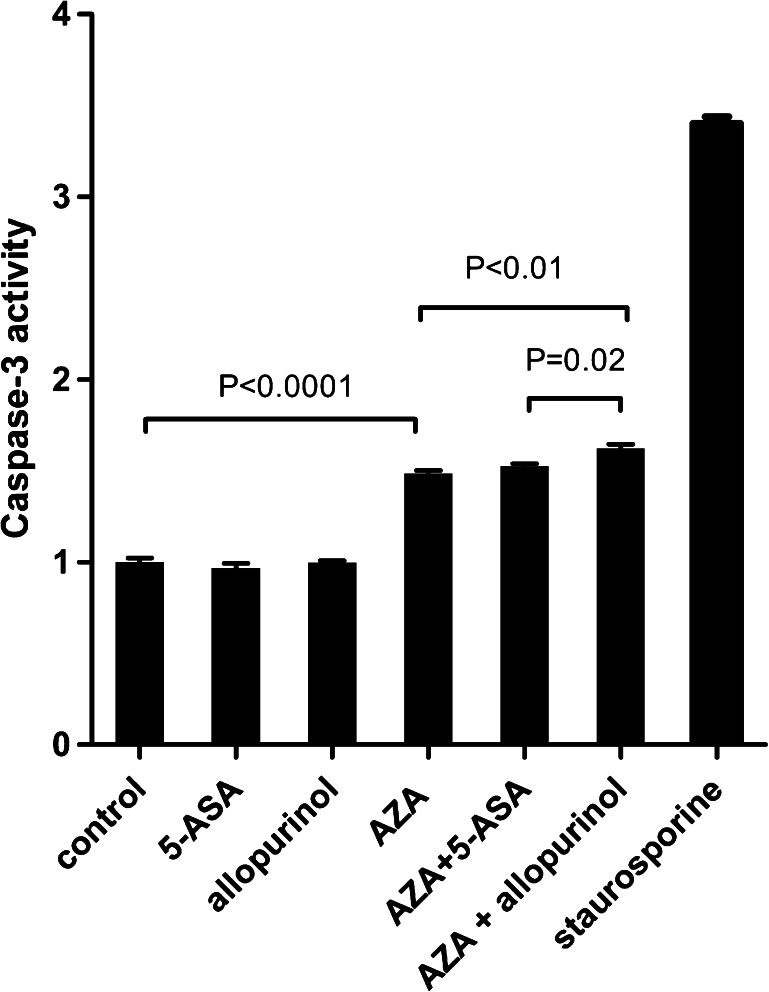
Caspase-3/7 activation of HepaRG cells incubated 24 h with medium (control), 5-ASA (200 μM), allopurinol (100 μM), AZA (70 μM) or a combination of both. Staurosporine (2 μM) was used as positive control. Activation of cells incubated only with medium was set as control. Values are presented as mean of two experiments with the SEM. Differences were compared with the Mann–Whitney *U* test. *AZA* azathioprine, *5-ASA* 5-aminosalicylic acid, *SEM* standard error of the mean

DNA damage by AZA with or without allopurinol was visualised with single cell electrophoreses (Fig. 6). With levels of AZA (70 μM) not affecting cell viability, the combination of AZA + allopurinol resulted in larger comet tails (more DNA damage) than incubation with AZA alone. In addition, caspase-3/7 activation was significantly increased (*P* < 0.01) when allopurinol (100 μM) was co-administered with AZA (70 μM), while allopurinol alone did not increase caspase-3/7 activation (Fig. 7). Co-administration of 5-ASA (200 μM) with AZA (70 μM) did not influence caspase-3/7 activation (*P* = 0.17).

## Discussion

In this study, we showed that the combination of a non-toxic dose of 100 μM allopurinol with AZA, 6-MP or TG led to a twofold to threefold increase in thiopurine-induced cytotoxicity in HepaRG cells. The most likely explanation for the increased cytotoxicity with allopurinol is an elevation of 6-TGN levels, which are incorporated into DNA as fraudulent bases, resulting in strand breakage and apoptosis (Sahasranaman et al. 2008).

Previous studies in cultures of rat hepatocytes showed a positive effect on cell viability when allopurinol was co-administered with AZA, due to its anti-oxidant capacities (Lee and Farrell 2001; Tapner et al. 2004; Al Maruf et al. 2014). The contrasting findings of our study might be explained by differences in the experimental settings. Isolated rat hepatocytes exert no mitotic activity in contrast to human hepatoma cells. Because 6-TGNs act through incorporation into the DNA, this effect may be more pronounced in mitotically active cells. We hypothesise that the higher levels of 6-TGNs with allopurinol co-administration are responsible for the increased cytotoxicity. This is supported by our findings that allopurinol co-administration resulted in more single-strand breakages and significant increased caspase-3/7 activation. In vivo, co-administration of allopurinol results in a significant reduction of 6-MMP metabolites and increase of 6-TGN metabolites (Hoentjen et al. 2013). Unfortunately, we were not able to analyse 6-TGN levels by HPLC, because of equal retention times of AZA, 6-MP and 6-TGN. A direct comparison of our in vitro study with in vivo situation is difficult because of low mitotic activity of human hepatocytes compared with our human hepatoma cells. However, HepaRG cells are known to have decreased mitotic activity when they are confluent, resembling the in vivo situation as best as possible. (Andersson et al. 2012).

Theoretically, addition of 5-ASA may lead to decreased hepatotoxicity in vivo by inhibition of TPMT, leading to a decline in 6-MMP levels (Szumlanski and Weinshilboum 1995; Dubinsky et al. 2000; de Graaf et al. 2010). However, there is much debate about the clinical significance of this interaction (Actis et al. 2009). The interaction between 5-ASA and thiopurines is non-competitive, and long-term 5-ASA therapy did not affect TPMT enzyme activity in a prospective study in CD patients (Szumlanski and Weinshilboum 1995; Dilger et al. 2007). Unfortunately, measurement of 5-ASA influences on TPMT activity is technically not feasible to our knowledge, because 5-ASA is removed in the washing steps (Dilger et al. 2007). In our in vitro study, the effects of 5-ASA on cytotoxicity were limited compared to allopurinol. This is supported by the finding that 5-ASA co-administration did not increase caspase-3/7 activation.

In this study, we showed that concentration-based TG is more cytotoxic than AZA or 6-MP, with IC_50_ values of 18 μM in HepaRG cells after 72-h incubation. This probably results from a direct conversion of 6-TG to 6-TGN, resulting in higher 6-TGN levels and less other metabolites. The increased cytotoxicity of TG with allopurinol co-administered can be explained by XO involvement in TG metabolism (Fig. 1). Our results of single-drug assessments of AZA and 6-MP in HepaRG cells are in accordance with those of Petit et al., who compared HepaRG cells with human hepatocytes (Petit et al. 2008). Furthermore, we showed a different toxicity profile of AZA and 6-MP in Huh7 and HepG2 cells. This might be explained by depletion of glutathione, caused by the conversion of AZA to 6-MP, as previously shown by Lee and Farrell (Lee and Farrell 2001). On the other hand, in HepaRG cells, this difference was not that prominent.

We measured both TPMT activity as well as the most prevalent polymorphisms in the *TPMT* gene (TPMT*2 and TPMT*3A, *3B), because TPMT activity still varies in patients without these polymorphisms (Lennard 2013). Low TPMT activity is associated with high 6-TGN levels, which may cause leucopenia (Shaye et al. 2007; Lennard 2013). The three hepatoma cell lines tested had identical *TPMT* genotypes, but a variance in TPMT activity was noticed. Hence, no direct relation with cytotoxicity was found. It is difficult to compare TPMT activity we measured with those in clinical studies as herein TPMT activity is measured in erythrocytes (Ford and Berg 2003).

Unfortunately, our HPLC method could not discriminate 6-TGN from AZA or 6-MP. This would be of particular interest, as that would allow the production of a dose response curve comparing the association between 6-TGN levels and cytotoxicity. Despite the fact that the used HPLC protocol was not intended and validated for cell line research, our study is still unique, providing direct evidence of the in vitro metabolism of AZA in the HepaRG cell line demonstrated by the formation of 6-MMP. Another limitation of this study is the lack of comparison with human hepatocytes. It has to be stressed that incubation experiments with human hepatocytes also have their limitations, as human hepatocytes are difficult to obtain and their metabolic activities rapidly decline during culturing (Gomez-Lechon et al. 2003; Guillouzo et al. 2007; Szabo et al. 2013). HepaRG cells are currently considered as the best surrogate for human hepatocytes for use in drug toxicity studies and are closer related to human hepatocytes than rat hepatocytes because of the high conservation of metabolic enzymes and the absence of interspecies differences (Parent et al. 2004; Guillouzo et al. 2007; Hart et al. 2010; Andersson et al. 2012; Szabo et al. 2013). With the notion that HepG2 and Huh7 cells have been used more extensively in in vitro experiments so far, we also used these cell lines for comparison (Guo et al. 2011). In accordance with previous studies (Petit et al. 2008; Hart et al. 2010; Andersson et al. 2012), we showed that HepaRG cells express the highest levels of important drug metabolising enzymes. In order to create similar experimental conditions for the three cell lines, we used the same culture medium for all three cell lines, which includes addition of 2 % DMSO according to the HepaRG culturing protocol (Gripon et al. 2002). DMSO is used to induce or maintain cell differentiation in HepaRG cells (Gripon et al. 2002; Narimatsu et al. 2006; Sainz and Chisari 2006; Choi et al. 2009). Because previous studies with HepG2 and Huh7 cells did not use DMSO, this can be considered as a limitation. However, our results demonstrate that the upregulation of drug metabolising enzymes by DMSO is not limited to HepaRG cells, results which are in accordance with those of Choi et al. (Choi et al. 2009).

In conclusion, in this in vitro study, we could demonstrate a considerable increase of thiopurine-induced cytotoxicity by co-treatment with a non-toxic dose of allopurinol.

## Abbreviations

5-ASA: 5-Aminosalicylic acid
6-MP: 6-Mercaptopurine
6-TGN: 6-Thioguanine nucleotide
AZA: Azathioprine
TG: Thioguanine
6-MMP: 6-Methylmercaptopurine
TMPT: Thiopurine-S-methyltransferase
XO: Xanthine oxidase
DMSO: Dimethyl sulfoxide
